# Correlation between visual scores and parathyroid function

**DOI:** 10.3389/fendo.2023.1217795

**Published:** 2023-06-29

**Authors:** Leyre Lorente-Poch, Maite de Miguel-Palacio, Joan Sancho-Insenser

**Affiliations:** ^1^ Endocrine Surgery Unit, General Surgery Department, Hospital del Mar, Parc de Salut Mar, Barcelona, Spain; ^2^ Pompeu Fabra University, Barcelona, Catalonia, Spain

**Keywords:** parathyroid identification, visual scores, discolored parathyroid glands, pre-ICG era, parathyroid insufficiency

## Abstract

This mini review summarizes the controversies regarding routine parathyroid identification reviews publications that assess visual scores to predict parathyroid function after thyroidectomy during the pre-ICG era.

## Introduction

The majority of surgeons advocate for systematic identification of the four parathyroid glands at their orthotopic position to avoid incidental excision and prevent parathyroid insufficiency. However, some authors believe that identification has no influence on postoperative parathyroid insufficiency, or may even have a negative impact.

Regarding visual scores before the implementation of Indocyanine-Green (ICG) and Near Infrared Autofluorescence (NIRAF), surgeons would mainly rely on the appearance and color of parathyroid glands, on maneuvers to check gland viability, and use magnifying lenses to predict and prevent parathyroid insufficiency.

## To identify or not to identify?

Both, American (1) and European (2) guidelines on postoperative hypoparathyroidism, published back in 2015, recommend identifying all four parathyroid glands and state that accidental excision is a predisposing risk factor for parathyroid insufficiency. Along the same lines, recent International guidelines (3) advise leaving all viable parathyroid *in situ* and avoiding parathyroid autotransplantation as much as possible. In contrast, the ATA Statement on Postoperative Hypoparathyroidism (4) considers identifying all four parathyroid glands to not be essential.

Several studies assessing the effect of inadvertent parathyroidectomy found no influence on postoperative hypocalcemia (5–8). Furthermore, in some cases, such as the study conducted by the Irish ENT group of Sheaham et al. (9), parathyroid identification was found to be risky. They prospectively assessed 126 patients who underwent total thyroidectomy and found that the more parathyroid glands they identified, the higher the prevalence of hypocalcemia (defined as serum calcium lower than 2 mmol/L). Additionally, the number of accidentally removed glands (with a prevalence of 9.5%) was not influenced by the number of parathyroids identified.

In contrast, there were two prominent classic multicentric studies, conducted in Germany and Sweden, that conclude that parathyroid identification is important. Thomusch et al. (10) prospectively assessed 5,846 patients who underwent a bilateral thyroidectomy in 45 centers and found that identifying less than two parathyroid glands carried a 1.4-fold risk of transient hypoparathyroidism and a fourfold risk of hypoparathyroidism 6 months after surgery. Nevertheless, no added benefit was found when the number of identified parathyroid glands moved from two to three. The retrospective study performed by Bergenfelz et al. (11) assessed 3,660 thyroidectomies from the Scandinavian Quality Register for Thyroid and Parathyroid Surgery. It showed that the fewer parathyroid glands identified intraoperatively, the higher the risk for hypoparathyroidism 6 months after surgery. Although both studies boasted large populations and performed a meticulous analysis, both were flawed by the lack of histologic confirmation of parathyroids in the specimen.

Lastly, a thorough review and meta-analysis (12) assessing the predictors of post-thyroidectomy hypocalcemia selected 115 studies and found that, from the very first excised parathyroid gland, it impacted postoperative hypocalcemia, being an independent predicting factor for transient hypocalcemia. Identifying less than two parathyroid glands was isolated as a predicting factor for hypoparathyroidism at 6 months. According to these findings, the authors advocated for routine identification of parathyroid glands to ensure preservation *in situ*.

In our group, we routinely aim to identify all four parathyroid glands based on the results of a retrospective study (13) assessing 442 patients with total thyroidectomy in which we found that identifying fewer parathyroid glands was associated with a higher prevalence of low and undetectable parathormone (PTH) after surgery.

## Does the appearance of parathyroid glands matter?

Some surgeons carry out an autotransplantation when the parathyroid gland turns discolored, assuming it is a sign of impaired viability.

To answer this query, a detailed study from Austria (14) assessed the discolor of parathyroid glands in 29 patients with total or near-total thyroidectomy. Promberger et al. divided the sample into three groups: A) all four parathyroid glands were well preserved, B) three or four discolored parathyroid glands, and, finally, C) two autotransplanted parathyroid glands.

They measured PTH before skin incision, intraoperatively, 3 hours after surgery, and on 1^st^, 2^nd^, 3^rd^, and 10-14 POD, and 6 months after surgery. They observed that group B experienced a significant decrease in PTH levels but quickly recovered within 3 days. The small sample size did not allow them to show the impact of discoloration on protracted hypoparathyroidism at 14 POD nor at 6 months after surgery. They concluded that the viability of parathyroid glands cannot be judged by their color at all, and therefore recommended preserving discolored parathyroid glands *in situ.* This study was also flawed by the lack of histologic confirmation.

In line with the previous group, a well-designed study conducted by Lang et al. (15) prospectively analyzed 103 patients with total thyroidectomy from a tertiary Chinese center excluding those who received an autotransplantation and those with parathyroid glands found in the specimen. They defined discolored parathyroid glands as severely bruised glands or darkened by >50% relative to their entire surface area before closing the wound. They divided the sample into 3 groups: 1) with all parathyroid glands well perfused, 2) one to two discolored parathyroid glands, and 3) patients with three or more discolored parathyroid glands.

They found only differences between groups 1 and 3: when three or more parathyroid glands were discolored, serum calcium dropped at 24 h from baseline and PTH 24 hours after surgery were significantly lower. Impairment in discolored parathyroid glands quickly recovered within 4–6 weeks. In their multivariate analysis, having more than three discolored parathyroid glands was isolated as an independent predicting factor for transient hypoparathyroidism (OR: 14, 95% CI 1.6–124; p = 0.018). However, one of every four patients who developed hypoparathyroidism at one year pertained to group 1, with all parathyroid glands showing good color. They concluded that normal-colored parathyroid glands with seemingly adequate blood supply do not always imply a functionally normal gland. These findings highlight the need for a real-time intraoperative method to assess parathyroid gland viability.

## Maneuvers to check the viability of parathyroid glands

An American case series study from Luke’s–Roosevelt Hospital in New York, summarizes all the maneuvers to check the viability of parathyroid glands in 100 patients with total thyroidectomy for both benign and malignant cases (16). When they found a discolored parathyroid, they teased off the capsule to release any subcapsular hematoma. If the parathyroid did not recover, they applied 2 mL of 2% lidocaine since it has a spasmolytic effect on parathyroid gland vascularization. This would allow brisk bleeding when pricked with a 25-gauge needle in situations where the parathyroid initially appeared to have been completely devascularized. If the prick test was negative, they eventually autotransplanted the gland. With this technique, they claim they achieved 0 hypoparathyroidism at 1 month after surgery.

## Do the loupes help?

Not only identifying but magnifying the parathyroid glands may help to preserve them. An Italian study (17) showed a better performance with magnification loupes compared with a historical series in a total of 244 patients with total thyroidectomy performed for both benign and malignant conditions. In addition, to decrease inadvertent excision, operating with loupes significantly decreased both biochemical (20,6% *vs*. 33.9%; P=0.028) and clinical hypocalcemia (12.7% *vs*. 33%; P<0.001).

## Visual scores

To date, there are only two studies assessing naked-eye visual scores, both coming from Korea.

Kim et al. (18), retrospectively assessed a visual score in 316 patients who underwent total thyroidectomy and central neck dissection for thyroid carcinoma. The scoring ranged from 0 to 12 points per patient. When the parathyroid gland was discolored a patient received 1 point; when it was slightly discolored, 2 points; and when it was well preserved the patient received 3 points. They also divided the sample into three groups based on the duration of required calcium supplementation: group 1) received no supplementation beyond 2 weeks after surgery (82.3%), group 2 received supplements within 6 months after surgery (12%), and group 3 needed medication beyond 6 months (5.7%). The mean parathyroid score showed a significant inverse association with the duration of required calcium supplementation (Group 1: 7.32, Group 2: 6.45, Group 3:4.89; P < 0.001).

The authors claimed that parathyroid scores were positively correlated with ionized PTH concentrations at 2 hours, 2 weeks, and 3 months and that the correlation was even stronger at 6 months and 1 year after thyroidectomy. Nevertheless, all R values were lower than 0.1, therefore statistical correlation cannot be assumed. Although well-intentioned, this study was flawed by some limitations: it was not well established whether a score of 0 applied to accidental excision or autotransplantation. There was no information regarding the 20 patients who underwent autotransplantation or which score they received, and, additionally, there was no pathology confirmation.

Park et al. (19) conducted a prospective study assessing 209 patients with papillary carcinoma. This time the score was more detailed (2, intact color; 1.5, mild discoloration; 1.2, not identified; 1, autotransplantation; -1= sacrificed). They found that the mean of the score was significantly lower in patients with low PTH and those who developed transient hypocalcemia. That was not the case for permanent hypoparathyroidism. With this score, they were able to predict 87% of postoperative hypocalcemia. In light of these results, they proposed an algorithm to prescribe supplements based on postoperative PTH levels (< 14pg/mL) and on the parathyroid score with a cut-off < 6.3.

## Discussion

Since total thyroidectomy gained popularity in the late 1980s, postoperative hypocalcemia emerged as the most common complication after thyroid surgery, occasionally progressing to permanent hypoparathyroidism, a condition requiring life-long medical supervision and treatment.

There is a general consensus that the main cause of hypocalcemia after total thyroidectomy is acute parathyroid insufficiency. This results from intraoperative damage to the parathyroid glands through a combination of mechanical or thermal trauma, gland devascularization, and/or inadvertent removal, all leading to a transient or permanent reduction of the functional parathyroid parenchyma.

The majority of surgeons consider that failure to identify the parathyroid glands during thyroidectomy may increase the risk of injury and inadvertent excision. There are, however, some authors that found no effect of parathyroid identification on reducing accidental parathyroidectomy. The majority of these studies are retrospective and appear to be flawed by the inclusion of conservative surgical procedures and small sample sizes.

Our group assessed the importance of the number of parathyroid glands remaining *in situ* (PGRIS) (20) calculated using the formula: 4 minus (parathyroid glands autografted +parathyroid glands found in the specimen). The multivariable analysis of 669 total thyroidectomies showed that PGRIS was the most powerful predictive variable of hypocalcemia and hypoparathyroidism, both protracted (one month after surgery) and permanent (one year postoperatively). Inadvertent parathyroidectomy, a clue for a lower PGRIS score, is a crucial factor involved in parathyroid insufficiency. Efforts should be made to preserve the parathyroid glands *in situ*. In the same study, among patients with all parathyroid glands *in situ* (PGRIS=4), the prevalence of permanent hypoparathyroidism was 2.6%. Therefore, parathyroid ischemia plays a paramount role, and ICG angiography may help us to avoid the devascularization of parathyroid glands (21).

Regarding visual scores, there are few publications that assess the capacity to predict postoperative hypoparathyroidism. The results were good but nowadays have been overcome by ICG and NIRAF.

The seminal study conducted by Fortuny et al. in which they prospectively analyzed 36 patients with ICG scores showed excellent results in both the ICG and non-ICG groups (22).

In the group of ICG patients, they conducted an interesting analysis, showing that surgeons with the naked eye were significantly more optimistic than the ICG score when assessing parathyroid gland viability. Although both scores are subjective, it seems that the ICG score is more realistic.

In conclusion, we recommend the identification of parathyroid glands to avoid accidental excision and therefore, prevent parathyroid insufficiency. There is no data suggesting that discolored parathyroids play a role in long-term hypoparathyroidism and parathyroid visual scores with the naked eye may predict postoperative hypoparathyroidism but emerging data assessing ICG and NIRAF show more encouraging results.

## Author contributions

LL-P and JS-I contributed to the conception and design of the study. LL-P wrote the first draft of the manuscript. MM-P and JS-I wrote sections of the manuscript. All authors contributed to the manuscript revision, and read, and approved the submitted version.
